# The effectiveness of a workplace violence prevention strategy based on situational prevention theory for nurses in managing violent situations: a quasi-experimental study

**DOI:** 10.1186/s12913-023-10188-1

**Published:** 2023-10-26

**Authors:** Jianzheng Cai, Sisi Wu, Haifang Wang, Xiaoqing Zhao, Yajie Ying, Yingying Zhang, Zhaofang Tang

**Affiliations:** 1 Department of Nursing, the First Affiliated Hospital of Soochow University, Suzhou, 215006 China; 2 Medical Branch, Shanghai Jiao Tong University Press, Shanghai, 200030 China; 3 Department of Nursing, the First Affiliated Hospital of USTC, Hefei, 230036 China; 4 Department of Emergency, the First Affiliated Hospital of Soochow University, Suzhou, 215006 China

**Keywords:** Workplace Violence, Nurse, Situational prevention theory, Prevention strategy, Training

## Abstract

**Background:**

Workplace violence (WPV) poses a significant occupational hazard for nurses. The efficacy of current education and training programs in mitigating WPV incidence among nurses remains uncertain, possibly due to insufficient consideration of clinical contexts and nurses’ specific needs. Therefore, this study developed a WPV prevention strategy based on the actual requirements of clinical nurses and situational prevention theory and aimed to explore its application effects.

**Methods:**

Under the guidance of situational prevention theory, a WPV prevention strategy for nurses was constructed through literature review, semi-structured interviews and focus group discussion. This study adopted a self-controlled research design, and trained 130 nurses selected from a comprehensive tertiary grade A hospital in Suzhou in this WPV prevention strategy. Data were collected through structured questionnaires, including the revised WPV questionnaire, WPV severity grading scale, and hospital WPV coping resources scale. The WPV incidence, severity, and WPV coping resource scores of nurses were collected before the intervention, as well as at 3 months, 6 months, and 9 months after training.

**Results:**

The WPV prevention strategy comprised 11 prevention plans based on 11 high-risk situational elements of WPV. Each prevention plan included the WPV prevention flowchart, treatment principle, and communication strategy. The strategy demonstrated excellent feasibility and practicality. Following the intervention, the overall incidence of WPV among nurses significantly decreased from 63.85% (baseline) to 46.15% (9 months after training) (*P* < 0.05). After the training, the severity of psychological violence (Wald χ² = 20.066, *P* < 0.001) and physical violence (Wald χ² = 9.100, *P* = 0.028) reported by nurses decreased compared to the baseline (*P* < 0.05). Moreover, the overall WPV coping resource score significantly increased from [66.50 (57.00, 77.25) points] (baseline) to [80.00 (68.00, 97.25) points] (9 months after training) (*P* < 0.05).

**Conclusions:**

The described WPV prevention strategy, grounded in situational prevention theory and tailored to the needs of clinical nurses, effectively reduced WPV incidence, mitigated its severity, and enhanced nurses’ WPV coping resources. This approach offered new avenues for nurses in the prevention of WPV.

## Background

Workplace violence (WPV) within hospital settings has emerged as a significant global public health concern [1–4]. WPV can be categorized into two primary sources: internal and external. Internal violence refers to violence inflicted on medical staff by colleagues or superiors, while external violence involves violence directed towards medical staff by patients and their visitors [5]. Among healthcare professionals, nurses are particularly susceptible to external violence due to the nature of their work, which often involves extended interactions with patients [6–9]. This study specifically examined external WPV experienced by nurses. Nurses subjected to WPV may endure not only physical discomfort, such as chest tightness and headaches, but also psychological repercussions, such as fear, anxiety, and depression. These physical and psychological responses can lead to diminished job satisfaction, decreased work efficiency, and an increased turnover intention [10–13]. Shahjalal et al. have revealed that health workers’ absenteeism rate due to WPV-related injuries stands at 22.44% [14]. Moreover, a report commissioned by the American Hospital Association in 2017 has disclosed that hospitals incur substantial costs, amounting to $429 million, in medical expenses, staffing, and insurance expenses resulting from violent incidents against their staff [15]. It is evident that WPV not only disrupts the normal functioning of healthcare facilities but also places an economic burden on society. Hence, implementing measures to reduce WPV incidence among nurses is imperative. This action is crucial for enhancing nurses’ job satisfaction and well-being while simultaneously fostering the healthy development of the nursing profession.

After an extensive review of the literature, it has become evident that education and training have emerged as the most prevalent and comprehensive coping strategies for preventing and addressing WPV [16–19]. Numerous studies have further validated that education and training can significantly enhance nurses’ confidence in managing WPV and improve their ability to effectively handle such incidents [20–24]. For instance, a study by De et al. has demonstrated that nurses’ confidence in dealing with patient aggression significantly increases following behavior management training [25]. Similarly, Krull et al. have discovered that computer-based and simulation training, which emphasizes de-escalation techniques and restraint application, leads to enhanced staff perceptions of their knowledge, skills, confidence, abilities, and preparedness when managing violent patient behaviors [26]. However, the effectiveness of education and training in effectively reducing the incidence of WPV among nurses remains an area warranting further investigation [18, 27–30]. Deans et al. have provided de-escalation skills training for emergency nurses, but the results indicate that the incidence of WPV does not exhibit a significant decrease within 3 months after the intervention [29]. Fernandes et al. have reported a slight increase in the incidence of WPV among nurses at the 6-month mark post-intervention when compared to pre-intervention levels [30]. Therefore, it is of paramount importance to delve into the underlying reasons behind the limited effectiveness of education and training programs in reducing WPV incidence among nurses. Additionally, it is crucial to develop targeted WPV prevention strategies that can effectively reduce WPV incidence among nurses.

Presently, education and training programs aimed at preventing WPV predominantly encompass theoretical knowledge about WPV and practical skills training, such as de-escalation skills and breakaway techniques [24, 26, 27]. Some researchers have put forward reasons for the limited success of current education and training initiatives in reducing WPV incidents among nurses. For instance, Heckemann et al. have argued that these strategies cannot effectively prevent WPV, which may be linked to their broad content [31]. Additionally, Tolli et al. have suggested that the lack of efficacy can be attributed to training content that does not align with the actual needs of clinical nurses and a lack of theoretical underpinning in the construction of prevention strategies [32]. Furthermore, the current prevention strategies often neglect the specific clinical contexts in which violent incidents occur, resulting in suboptimal applicability and practicality in clinical settings. Therefore, it is essential to formulate targeted WPV prevention strategies, guided by scientific theories, that focus on high-risk situational elements of WPV and are tailored to the specific needs of clinical nurses. This approach may hold the key to reducing WPV incidence among nurses effectively.

According to the general emotion-aggression theory, situational factors serve as triggers for WPV against nurses [33]. Wolf has emphasized the importance of analyzing the situational elements of violent events as a means for nurses to comprehend the precursors of WPV and proactively identify potential WPV incidents [34]. Building upon this perspective, the project team initially adopted a mixed research design and identified 11 high-risk situational elements associated with WPV against nurses [35]. Furthermore, our project team conducted in-depth interviews with 18 nurses who both experienced WPV and received WPV training. The findings illuminated that the nurses were more familiar with the context of WPV in the process of violence prevention. A procedural, step-by-step WPV coping strategy emerged as better aligned with the practical needs of clinical nurses. In summary, existing WPV prevention strategies have often overlooked the role of the specific situation in promptly averting violent incidents. The broad scope of existing training content, coupled with limited clinical applicability, may explain their limited effectiveness in reducing WPV incidence among nurses. Therefore, formulating a step-by-step prevention strategy grounded in the high-risk situational elements of WPV is a promising approach to enhancing the effectiveness of WPV prevention for nurses.

Situational prevention theory places a strong emphasis on the context in which events occur, asserting that events are inherently intertwined with their surrounding circumstances [36]. According to this theory, both individuals and organizations have the capacity to influence the relevant elements of a situation. This can be achieved by making the occurrence of an event more challenging, increasing the risks of implementing the event, reducing the benefits for the implementer, minimizing the verbal and behavioral stimuli to the implementer, and eliminating excuses for implementing the event. Furthermore, it is advantageous to alter the developmental trajectory of an event as a means of prevention and response.

Therefore, this study aimed to formulate a step-by-step WPV prevention strategy for nurses under the guidance of the situational prevention theory and rooted in the 11 high-risk situational elements of WPV identified by the project team in earlier stages, and to explore the preliminary application effects of the prevention strategy to provide a basis for reducing the incidence of WPV among nurses.

## Methods

### Construction of the WPV prevention strategy for nurses

#### Preliminary construction of the WPV prevention strategy

The research team was comprised of two postgraduate tutors, five postgraduate students, and three clinical nurses. Prior to commencing the study, team members conducted a comprehensive literature review to acquire information related to WPV education and training, and learned relevant theoretical knowledge and operational skills. In line with the situational prevention theory, which emphasizes that situations can be “modified” through specific measures, including increasing the difficulty of violence occurrence, increasing the risk of committing violence, reducing the benefits for perpetrators, minimizing verbal and behavioral stimuli to perpetrators, and eliminating excuses for committing violence. Accordingly, the project team formed an interview outline based on the countermeasures provided by situational prevention theory and the 11 high-risk situational elements of WPV among nurses (Table 1) which were summarized by the project team at an early stage.

From September 2020 to March 2021, individuals with firsthand experience of WPV, including nurses and managers who dealt with WPV incidents, were selected through purposive sampling for semi-structured interviews. During this phase, the prevention strategy was initially formulated, encompassing both verbal and behavioral components. The specific interview outline included the following questions: (1) What do you think nurses should do first when encountering the high-risk situation of WPV? (2) What actions and words should nurses employ to make violence occurrence more challenging? (3) What actions and words should nurses employ to increase the risk of committing violence? (4) What actions and words should nurses employ to reducing the benefits for perpetrators? (5) How can nurses minimize verbal and behavioral stimuli to perpetrators? (6) What actions and words should nurses employ to eliminate excuses for committing violence? (7) What measures should nurses adopt to ensure their personal safety? (8) Do nurses need to seek assistance in such situations? If so, who is the appropriate person to approach, and how can nurses effectively request help in a reasonable manner?

**Table 1:** 11 High-risk situational elements of WPV among nurses

Core Category	Category	Core Category	Category
Antecedent state elements	When the patient is an alcoholic	Environmental or Institutional elements	When dissatisfied with the hospital environment
	When the patient’s mental state is abnormal		When dissatisfied with the hospital treatment system or process
Purpose elements	When the doctors or nurses were not found repeatedly	Timing elements	When dissatisfied with the nurse’s service attitude
	When the waiting time was long		When questioning medical expenses
	When the unreasonable request was refused		When refusing to accept the diagnosis of the current condition
			When receiving an invasive operation unsuccessfully for the first time

#### Development of the final draft of the WPV prevention strategy

The final draft of the WPV prevention strategy was crafted through a series of focus group discussion. These discussions involved in-depth deliberations by focus group members on the scientific rigor, feasibility of the content, and the rationality of the process presented in the initial draft of the prevention strategy. The goal was to arrive at a consensus regarding the definitive content of the WPV prevention strategy for nurses. To ensure the strategy’s applicability across diverse clinical contexts, the study took into consideration the comprehensiveness and diversity of the focus group members.

A total of seven rounds of focus group discussion were conducted, comprising two distinct parts. Firstly, experts from various fields, such as psychology and law, were selected for two rounds of deliberation. Secondly, nurses and nursing managers with extensive experience in WPV prevention and response, hailing from different departments in primary hospitals, secondary hospitals, tertiary hospitals, children’s specialized hospitals, and psychiatric hospitals, were selected as focus group members for five rounds of discussion. This multifaceted approach aimed to render the prevention strategy more versatile for application within clinical settings. The inclusion criteria for experts participating in focus group discussion were as follows: ① nurses with extensive experience in WPV prevention and response, nursing managers, security department personnel, patient service center staff, medical office personnel, doctor-patient communication office staff, psychologist, legal experts and other relevant personnel; ② Bachelor’s degree or higher; ③ A minimum of 10 years of professional experience; ④ Informed consent and voluntary participation in the study.

### Preliminary application of the prevention strategy

#### Participant recruitment

From May 2021 to February 2022, nurses who met the inclusion criteria were selected as research subjects in a comprehensive tertiary grade A hospital in Suzhou using convenience sampling. The recruitment process followed these steps. Prior to the study commencement, a notice was posted on the hospital’s website inviting nurses to participate in the research. The notice included details about the research purpose, significance, training content, schedule, location, and relevant instructions. Nurses willing to participate could register in the designated conference room. The inclusion criteria for research subjects were as follows: clinical nurses engaged in direct patient contact as part of their daily responsibilities; a minimum of 1 year of experience in clinical nursing; and informed consent and voluntary participation in the study. The exclusion criteria were as follows: nurses on leave during the survey period, including maternity and sick leave; and refresher nurses.

This study received approval from the hospital’s ethics committee, and informed consent was obtained from the participating nurses through the signing of informed consent forms before the study commenced.

#### Intervention

The research subjects underwent an intervention involving the application of the WPV prevention strategy rooted in situational prevention theory. This intervention was carried out by nurses who possessed extensive knowledge of WPV, substantial experience in managing WPV incidents, and held senior professional titles. The intervention method comprised a combination of classroom teaching and scene simulation training conducted over a duration of 10 months. Detailed information about the intervention plan can be found in Table 2.

**Table 2:** Content of the intervention plan

Time Point		Interventions
Pre-intervention		Before the formal start of the intervention, the researchers communicated with the functional departments of the hospital, coordinated relevant issues among the hospital management organization, departments and nurses, and issued a notice in advance to inform the participants of the time, place and content of the training.
Intervention	Week 1 (6 lessons, 360 min)	①Before the first training, the researchers provided unified guidance to the nurses to fill in the questionnaires, and collected their baseline data. The WPV prevention strategy training manual was distributed to each nurse. ② Classroom teaching: theoretical knowledge of WPV, the WPV prevention strategy of antecedent state elements
	Week 2 (6 lessons, 360 min)	① Classroom teaching: the WPV prevention strategy of environmental or institutional elements ② Scene simulation training: the WPV prevention strategy of antecedent state elements and environmental or institutional elements
	Week 3 (6 lessons, 360 min)	① Classroom teaching: the WPV prevention strategy of purpose elements ② Scene simulation training: the WPV prevention strategy of purpose elements and timing elements
	Week 4 (8 lessons, 480 min)	① Classroom teaching: the WPV prevention strategy of timing elements ② Summary: The researchers summarized the knowledge taught, reviewed the deficiencies in the scene simulation training, and responded to the participants’ questions. A diary was distributed to each nurse so they could record WPV incidents, and guidance was provided to nurses to fill in said diary correctly.
Post-intervention		At 3, 6 and 9 months after the training, questionnaires were distributed and collected to evaluate the preliminary application effects of the prevention strategy.

#### Instruments

##### The revised WPV questionnaire

Designed by Chen et al. [37], the first section of this questionnaire was employed in this study to investigate the occurrence of WPV among nurses. It consisted of four WPV items: verbal aggression, verbal threats, physical aggression, and sexual harassment. The test-retest reliability of the scale, as measured by the project team, was 0.803, and the average validity of each item was 0.916.

##### WPV severity grading scale

Zhu et al. [38] have developed this scale based on criminal law and other relevant legal standards. It is primarily used to assess the severity of WPV. The assessment criteria included several items as follows. Severity grading of physical violence: level 0 signifies no physical violence; level 1 represents physical attacks on medical staff without causing injury; level 2 encompasses physical violence leading to minor injuries to medical staff; level 3 entails physical violence resulting in moderate injuries to medical staff; and level 4 denotes physical violence resulting in severe injuries to medical staff.

Severity grading of psychological violence: level 0 corresponds to no psychological violence; level 1 signifies no impact; level 2 encompasses psychological violence resulting in effects, such as depression but not preventing medical staff from working; level 3 includes psychological violence with a substantial impact on the well-being of medical staff, causing them to temporarily cease working; and level 4 involves psychological violence leading to serious consequences, such as severe anxiety and insomnia, necessitating psychological intervention.

##### Hospital WPV coping resources scale

Compiled by Wang [39], this scale assesses the level of WPV coping resources available to nurses. It comprises 20 items across four dimensions, reflecting the subjects’ cognition, anticipation, organizational support, and coping abilities in relation to WPV. Each item is scored on a scale of 1 to 6, and the cumulative score for all items yields the total score for the scale. Scores range from 20 to 49 points for a low level, 50 to 89 points for a medium level, and 90 to 120 points for a high level of coping resources. The scale exhibits strong reliability and validity, with a Cronbach’s alpha coefficient of 0.920.

#### Data collection

Questionnaires was used for data collection. Prior to the first intervention, the researcher provided a comprehensive overview of the study’s purpose, methods, and relevant considerations to the nurses. Informed consent was obtained from each nurse, after which questionnaires were distributed. The research team members provided consistent guidance to assist the nurses in completing the questionnaires. Once the questionnaires were completed, the research team members immediately checked them on the spot, and immediately verified the missing items and uncertainties with the nurses who filled in the questionnaires.

#### Data analysis

Data analysis was performed using SPSS 22.0. Enumeration data were described in terms of frequency and composition ratio (%). If the measurement data adhered to a normal distribution, the mean and standard deviation were employed for description (${\bar x}$±S). In cases where the measurement data did not follow a normal distribution, the median and interquartile range were used for description M (P_25_, P_75_). Measurement data that conformed to both normal distribution and homogeneity of variance were compared using repeated measures ANOVA. Enumeration and measurement data that did not adhere to normal distribution or homogeneity of variance were analyzed using a generalized estimating equation. Pairwise comparisons of enumeration data at different time points were assessed using a Chi-square test or rank sum test. In this study, a significance level of *P* < 0.05 was applied to denote statistical significance.

## Results

### Content of the WPV prevention strategy

The final content of the WPV prevention strategy was determined through a comprehensive process involving seven rounds of focus group discussion, which included a total of 70 experts. The experts’ ages ranged from 34 to 54 years (with a mean age of 39.90 ± 5.95 years), and their professional experience spanned from 11 to 34 years (with a mean experience of 17.60 ± 6.90 years). All experts held senior professional titles, and 60% of them possessed a master’s degree or higher. The analysis indicated that the experts had a high level of average familiarity (Cs = 0.86), judgment basis (Ca = 0.90), and group authority coefficient (0.88), all of which exceeded the threshold of 0.70, signifying the reliability of the focus group discussion outcomes.

**Fig. 1:**
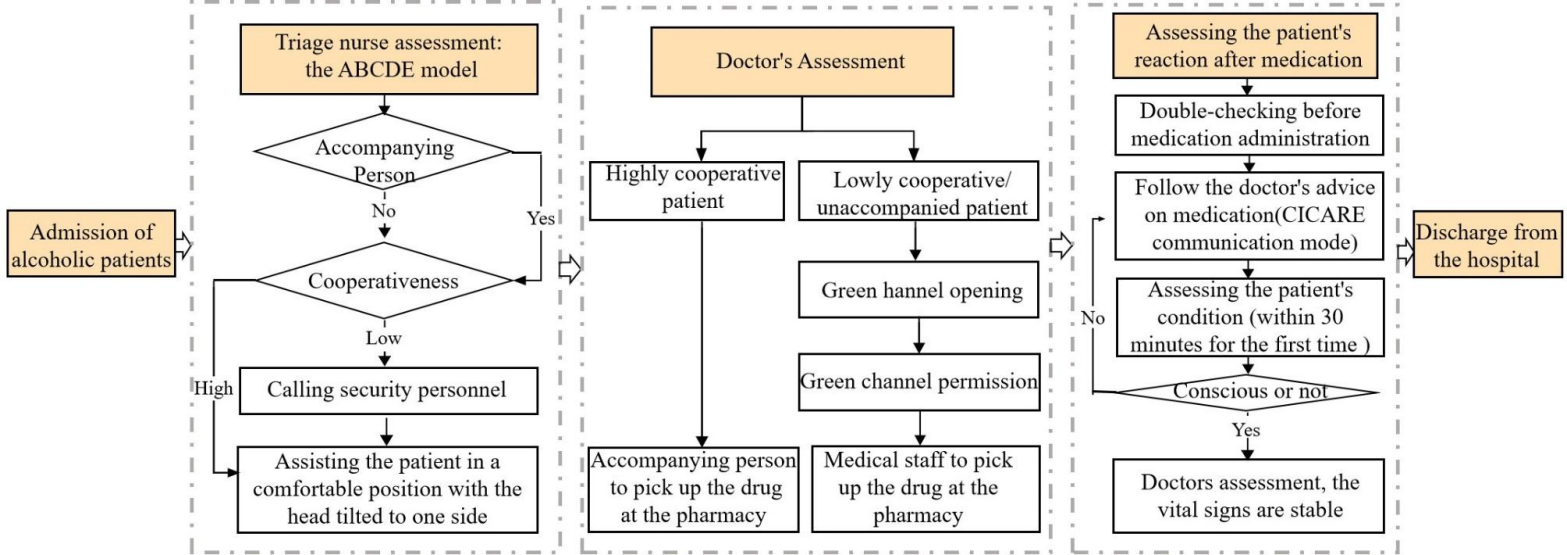
WPV prevention flowchart for nurses encountering “alcoholic patients”

The WPV prevention strategy comprised 11 prevention plans, each corresponding to one of the 11 high-risk situational elements of WPV. Each prevention plan featured the prevention flowchart, handling principle, and communication strategy based on the CICARE communication mode (Connect, Introduce, Communicate, Ask, Respond, Exit). The following content illustrates the WPV prevention strategy designed for nurses dealing with “alcoholic patients”, including the prevention flowchart (Fig. 1), handling principle, and communication strategy. It serves as a representative example of all strategies, which share a uniform construction approach.

#### Handling principle

Communicate with the patient’s family members, providing them with information regarding the potential effects of alcoholism on the patient’s current condition, the expected response to medication, and the subsequent follow-up treatment. Avoid offering additional information unless they inquire to prevent unnecessary disputes from arising.

### CICARE communication strategy

C: Hello to the family of XXX patient! (If there is no family member of the patient, please ask the security guards or colleagues to assist in taking the proper position for the nursing operation.)

I: I’m the nurse in charge of the patient. My name is XXX.

C: The patient has lost consciousness due to excessive alcohol intake, which may lead to adverse reactions such as vomiting. To prevent the patient from accidentally inhaling vomit into the airway and risking asphyxiation, I will gently turn the patient’s head to one side. Your cooperation in this matter is greatly appreciated.

A: To expedite the patient’s recovery and help the patient regain consciousness as soon as possible, it is essential to administer infusion therapy in accordance with the medical prescription. Is that OK?

R: Respond and give feedback to questions from patients and their family members (ask for help from colleagues or nurse managers if necessary).

E: Please assist me in closely monitoring the patient’s condition during this period. If any symptoms, such as restlessness, become evident in the patient, kindly notify me promptly. Additionally, I will make regular visits to reevaluate the patient’s status.

### Nurses’ general information

A total of 130 nurses were included in this study, with no dropouts observed at any time point before or after the intervention. The nurses’ ages ranged from 23 to 53 years, with an average age of (34.76 ± 6.65) years. The distribution of nurses across departments was as follows: emergency department (13.85%), outpatient department (14.62%), surgery department (32.31%), internal medicine department (20.77%), obstetrics and gynecology department (11.53%), and ICU (6.92%).

### Comparison of the incidence of WPV in nurses

The survey results indicated that the overall incidence of WPV experienced by nurses exhibited a statistically significant time effect (*P* < 0.05). Furthermore, the overall incidence of WPV, as well as the incidences of verbal aggression and verbal sexual harassment at 3 months, 6 months, and 9 months after the training, demonstrated a significant decrease compared to the baseline (*P* < 0.05). Detailed data are presented in Table 3.

**Table 3:** Comparison of the frequency and percentage of WPV occurrence in nurses

Time Point		Total violence	Verbal aggression	Verbal threat	Verbal sexual harassment	Physical aggression	Physical sexual harassment
T_0_		83(63.85)	66(50.77)	41(31.54)	15(11.54)	11(8.46)	5(3.85)
T_1_		67(51.54)	47(36.15)	29(22.31)	5(3.85)	4(3.08)	3(2.31)
T_2_		62(47.69)	43(33.08)	22(16.92)	4(3.08)	4(3.08)	2(1.54)
T_3_		60(46.15)	40(30.77)	21(16.15)	3(2.31)	3(2.31)	2(1.54)
*Wald* χ^2^ (Time effect)		10.315	15.005	12.099	13.189	8.758	1.884
*P*		0.016	0.002	0.007	0.004	0.033	0.597
Multiple comparisons							
*P*	T_0_-T_1_	0.045	0.017	0.093	0.020	0.111	0.720
	T_0_-T_2_	0.009	0.004	0.006	0.017	0.111	0.443
	T_0_-T_3_	0.004	0.001	0.004	0.007	0.054	0.443
	T_1_-T_2_	0.535	0.602	0.274	0.734	1.000	0.652
	T_1_-T_3_	0.385	0.358	0.208	0.720	0.702	0.652
	T_2_-T_3_	0.804	0.690	0.867	0.702	0.702	1.000

Note: T_0_: before the intervention; T_1_: 3 months after the training; T_2_: 6 months after the training; T_3_: 9 months after the training

### Comparison of the severity of WPV in nurses

The severity of WPV experienced by nurses exhibited a statistically significant time effect, as indicated in Table 4 (*P* < 0.05). Pairwise comparisons of WPV severity at four-time points revealed a significant reduction in WPV severity among nurses at 3 months, 6 months, and 9 months after training compared to the baseline, with statistically significant differences (*P* < 0.05). Specific details of the comparison of WPV severity before and after the intervention are provided in Table 4.

**Table 4:** Comparison of the frequency and percentage of WPV severity in nurses

Time Point		Psychological violence					Physical violence				
		0	1	2	3	4	0	1	2	3	4
T_0_		48(36.92)	25(19.23)	53(41.41)	4(3.08)	0(0.00)	117(90.00)	10(7.69)	3(2.31)	0(0.00)	0(0.00)
T_1_		63(48.46)	23(17.69)	43(33.59)	1(0.77)	0(0.00)	125(96.15)	5(3.85)	0(0.00)	0(0.00)	0(0.00)
T_2_		70(53.85)	26(20.00)	34(26.56)	0(0.00)	0(0.00)	125(96.15)	5(3.85)	0(0.00)	0(0.00)	0(0.00)
T_3_		71(54.62)	32(24.61)	27(20.77)	0(0.00)	0(0.00)	126(96.92)	4(3.08)	0(0.00)	0(0.00)	0(0.00)
*Wald* χ^2^ (Time effect)		20.066					9.100				
*P*		<0.001					0.028				
Multiple comparisons											
*P*	T_0_-T_1_	0.043					0.048				
	T_0_-T_2_	0.001					0.048				
	T_0_-T_3_	<0.001					0.023				
	T_1_-T_2_	0.238					1.000				
	T_1_-T_3_	0.094					0.735				
	T_2_-T_3_	0.638					0.735				

Note: T_0_: baseline; T_1_: 3 months after the training; T_2_: 6 months after the training; T_3_: 9 months after the training

### Comparison of the coping resources levels of WPV in nurses

The scores related to WPV coping resources among nurses exhibited a statistically significant time effect, as detailed in Table 5 (*P* < 0.05). Pairwise comparisons of WPV coping resources at four-time points revealed improvements in both the overall scores and the scores for all dimensions of WPV coping resources at 3 months, 6 months, and 9 months after the training compared to before the intervention. Additionally, the comparison of nurses’ ability to anticipate and cope with WPV at 3 months and 9 months after the training demonstrated a statistically significant difference (*P* < 0.05). The comparisons of WPV coping resources before and after the intervention are presented in Table 5.

**Table 5:** Comparison of the median and interquartile range of WPV coping resources levels in nurses

Time Point		Total violence	Cognitive ability	Anticipation ability	Coping ability	Organizational Support
T_0_		66.50(57.00,77.25)	20.00(17.00,24.00)	15.50(14.00,19.00)	23.00(19.75,28.00)	7.00(6.00,8.00)
T_1_		79.00(65.00,92.00)	24.00(20.00,27.00)	18.50(15.00,23.00)	28.00(22.00,33.00)	8.00(7.75,10.00)
T_2_		80.00(71.75,98.00)	24.00(22.00,29.25)	20.00(16.75,24.00)	28.00(24.75,35.00)	9.00(8.00,10.00)
T_3_		80.00(68.00,97.25)	24.00(20.00,29.25)	20.00(16.00,25.00)	28.00(24.00,35.00)	9.00(7.00,10.00)
*Wald* χ^2^ (Time effect)		103.784	78.161	75.234	90.719	75.465
*P*		<0.001	<0.001	<0.001	<0.001	<0.001
Multiple comparisons						
*P*	T_0_-T_1_	<0.001	<0.001	<0.001	<0.001	<0.001
	T_0_-T_2_	<0.001	<0.001	<0.001	<0.001	<0.001
	T_0_-T_3_	<0.001	<0.001	<0.001	<0.001	<0.001
	T_1_-T_2_	0.104	0.101	0.131	0.134	0.230
	T_1_-T_3_	0.053	0.444	0.007	0.014	0.968
	T_2_-T_3_	0.882	0.366	0.669	0.754	0.326

Note: T_0_: baseline; T_1_: 3 months after the training; T_2_: 6 months after the training; T_3_: 9 months after the training

## Discussion

### Scientificity and feasibility of the prevention strategy

The development of the prevention strategy in this study was guided by the principles of situational prevention theory, which focuses on technical measures to modify the situation to prevent violence. The interview outline was designed based on the five key aspects of this theory: increasing the difficulty of violence occurrence, increasing the risk of committing violence, reducing the benefits for perpetrators, minimizing verbal and behavioral stimuli to perpetrators, and eliminating excuses for committing violence. After conducting semi-structured interviews, the initial draft of the prevention strategy was created. After conducting focus group discussion, further refinements were made based on the feedback from the focus group discussion, ultimately resulting in the final prevention strategy.

The application of situational prevention theory in managing WPV has been demonstrated in previous research [40], reinforcing its suitability for WPV prevention. Meanwhile, the average familiarity (Cs) of the experts who formed the focus group in this study was 0.86, the judgment basis (Ca) was 0.90, and the authority coefficient of the experts was high (0.88). The experts covered the fields of nursing management, doctor-patient communication, psychology, law and other fields, which provided a robust and scientifically sound framework for constructing the prevention strategy, ensuring its scientific rigor.

According to the path of occurrence of WPV, situational elements are the triggers of violence and are unique in the process of violence occurrence. Research has revealed that nurses are familiar with the situations that lead to WPV [35]. Therefore, this study specifically targeted the 11 high-risk situational elements of WPV among nurses and formulated a corresponding prevention strategy. This strategy encompassed 11 prevention plans, each addressing a specific high-risk situational element, and included comprehensive guidance in the form of prevention flowcharts, treatment principles, and communication strategies. The strategy’s step-by-step approach empowered clinical nurses with actionable and feasible measures to prevent and respond to WPV effectively, meeting their practical needs.

### Effects of the prevention strategy on the incidence and severity of WPV among nurses

This study developed and implemented the WPV prevention strategy based on situational prevention theory, providing training to clinical nurses over a 10-month period. The results demonstrated a significant reduction in the overall incidence of WPV among nurses, decreasing from 63.85 to 46.15%. Additionally, there was a decrease in the severity of both psychological and physical violence suffered by nurses. These findings indicated that the prevention strategy effectively reduced the incidence and severity of WPV among nurses, possibly by enhancing their ability to prevent WPV [41, 42].

The prevention strategy appeared to improve nurses’ capacity to detect potential violence threats promptly, thanks to its focus on high-risk situational elements of WPV. Nurses can proactively apply the corresponding strategies to prevent violence in specific situations, thereby averting its occurrence and escalation. As a result, the prevention strategy not only reduced the incidence of WPV but also mitigated its severity.

Analysis of the different types of WPV revealed that both verbal and physical violence incidences decreased after the intervention. The use of the CICARE communication mode in the prevention strategy likely contributed to improved nurse-patient communication, thereby reducing incidents of verbal violence stemming from inappropriate communication by nurses [28]. It is worth noting that verbal violence can serve as a precursor to physical aggression. When verbal violence is not effectively addressed, it can escalate into physical violence. Conversely, effective management of verbal violence can lead to a reduction in physical aggression [43]. Consequently, the prevention strategy effectively reduced the incidence of both verbal and physical violence.

However, it is important to note that the decline in the incidence of both verbal and physical violence tapered off and stabilized over time. This could be attributed to the diminishing impact of the initial training as nurses gradually forgot the content and lost some of the training stimulus. To maintain the long-term effectiveness of the prevention strategy, hospital administrators should consider conducting regular WPV training for nurses to reinforce their knowledge and skills, ultimately reducing the occurrence of violence.

### Effects of the prevention strategy on the WPV coping resources level of nurses

Coping resources, often referred to as stress buffer factors, encompass a range of measures used to alleviate stress and stress-related reactions. WPV is a common source of stress for nurses, and WPV coping resources can be categorized into four aspects: cognition, anticipation, coping ability, and organizational support [44]. The results of this study demonstrated a notable increase in the overall score of WPV coping resources, rising from 66.50 (57.00, 77.25) points to 80.00 (68.00, 97.25) points after the intervention. Furthermore, the overall score, as well as the scores in all dimensions of WPV coping resources at each time point, were significantly higher than those at baseline (*P* < 0.05). This finding suggested that the prevention strategy effectively enhanced nurses’ WPV coping resources.

Firstly, the prevention strategy incorporated theoretical knowledge about WPV, which improved nurses’ understanding of WPV following the intervention. This aligned with the findings of Abozaid et al. [45]. The improved cognitive understanding of WPV enables nurses to identify violent behaviors more effectively at an early stage. Secondly, previous WPV prevention strategies often focus on independent research related to WPV theory, risk assessment, and response measures [46–49]. However, WPV prevention should be a comprehensive and dynamic process that encompasses theory, identification, and response. Based on situational prevention theory, this study elucidated the entire process of WPV, covering occurrence, early warning identification, and countermeasure development by selecting high-risk situational elements of WPV and conducting targeted analyses. This approach not only facilitated nurses’ rapid and accurate identification of WPV but also enabled them to respond to violent incidents effectively. Consequently, nurses’ ability to cope with WPV significantly improved after training.

Additionally, as this study was conducted, the quantity and content of WPV education and training resources for nurses increased. Nurses’ awareness of WPV expanded gradually, and hospital managers paid greater attention to WPV [50]. Consequently, nurses received more pronounced organizational support, leading to an increase in organizational support scores. These findings provided a solid foundation for promoting and applying this prevention strategy in WPV education and training for clinical nurses.

## Limitations

Firstly, the WPV prevention strategy in this study was constructed using qualitative methods. During the implementation of the prevention strategy, nurses from only one hospital were selected for training. The limitations of research methods and sample selection might restrict the generalizability of the results of this study. In the future, multicenter and large-sample studies can be conducted in different regions to further validate the effectiveness of the described prevention strategy. Additionally, the content of the prevention strategy can be continuously refined and improved during its wider implementation.

Secondly, due to the impact of epidemic prevention measures, nursing human resources were in short supply at the time of this study. Consequently, the implementation process with multiple groups was complicated. This study aimed to verify the effectiveness of the prevention strategy, which is why a self-controlled research method was adopted. In future studies, it is recommended to use randomized controlled trials for study design, which can provide stronger evidence for the effectiveness of the strategy.

Furthermore, due to the limited training time provided in this study, it may have potential implications for nurses’ performance in dealing with violence. In future research, it is necessary to organize regular training sessions for nurses and explore the use of online training platforms to facilitate ongoing learning. This will help nurses continually refine their skills and consolidate the training effects.

## Conclusions

In summary, while education and training are commonly used strategies for preventing and addressing WPV among nurses, the effectiveness of existing programs in reducing WPV incidence remains uncertain. This study, guided by the actual needs of clinical nurses and situational prevention theory, developed a WPV prevention strategy for nurses. The results demonstrated the efficacy of this strategy in reducing WPV incidence, decreasing the severity of WPV, and enhancing nurses’ coping resources for WPV. The strategy proved to be feasible and practical for implementation in clinical settings.

It is important to note that the underreporting of WPV incidents by nurses may lead to an inaccurate understanding of the true prevalence of WPV and an inaccurate assessment of intervention effectiveness. Therefore, in future research, it is essential to investigate the factors that influence nurses’ reporting of WPV and take measures to encourage nurses to report violent incidents more willingly. This will contribute to a comprehensive understanding of WPV occurrences and facilitate more effective measures to reduce its incidence.

## Data Availability

The datasets used in the current study are available upon reasonable request from the corresponding author.
